# Enzymatically Modified Isoquercitrin: Production, Metabolism, Bioavailability, Toxicity, Pharmacology, and Related Molecular Mechanisms

**DOI:** 10.3390/ijms232314784

**Published:** 2022-11-26

**Authors:** Aleksandra Owczarek-Januszkiewicz, Anna Magiera, Monika Anna Olszewska

**Affiliations:** Department of Pharmacognosy, Faculty of Pharmacy, Medical University of Lodz, 1 Muszynskiego St., 90-151 Lodz, Poland

**Keywords:** isoquercitrin oligoglucosides, AGIQ, flavonols, ADME, absorption, anti-inflammatory, cardioprotective, chemopreventive, neuroprotective, musculotropic

## Abstract

Quercetin and its glycosides, such as isoquercitrin or rutin, are among the most ubiquitous flavonoids present in plants. They possess numerous health-promoting properties, whose applicability is, however, limited by poor water solubility and absorption issues. Enzymatically modified isoquercitrin (EMIQ) is an isoquercitrin derivative obtained from rutin via enzymatic transformations that greatly enhance its bioavailability. Due to advantageous reports on its safety and bioactivity, EMIQ is currently gaining importance as a food additive and a constituent of dietary supplements. This review summarizes the thus-far-conducted investigations into the metabolism, toxicity, biological properties, and molecular mechanisms of EMIQ and presents a comprehensive characterization of this valuable substance, which might represent the future of flavonoid supplementation.

## 1. Introduction

Flavonoids represent a large group of plant polyphenols, with over 10,000 compounds identified until now. Based on the differences in the basic structure, they might be subdivided into several subclasses, such as flavonols, flavan-3-ols, flavanones, flavones, isoflavones, chalcones, and biflavonoids. In recent years, there has been growing interest in flavonoids, particularly flavonols, due to the increasing evidence for their prominent role in the prevention and treatment of various human diseases. Quercetin (3,3′,4′,5,7-pentahydroxyflavone; QU) is the best-known, prototypical representative of the flavonol subclass, which is among the most-widespread and most-studied types of flavonoids. Plants contain QU not only as an aglycone, but also in various conjugated forms such as glycosides, among which isoquercitrin (quercetin 3-*O*-β-d-glucopyranoside; IQ) and rutin (quercetin 3-*O*-β-d-rutinoside; RT) are the most ubiquitous [1,2]. These compounds are the main health-promoting components of black elderberry, capers, cloves, black chokeberry, grapes, buckwheat, onions, and olives, among others [3,4]. Accumulating in vitro and in vivo data suggest that QU and its glycosides have numerous beneficial properties, including antioxidant, cytoprotective, vasoprotective, anticarcinogenic, neuroprotective, anti-inflammatory, and antidiabetic effects [3,5,6,7,8]. However, their low solubility in water and other hydrophilic media, resulting in poor bioavailability [9], severely limits the potency of their positive health effects and applicability in medicine, pharmacy, and the food industry. Therefore, improving the water solubility of flavonols without compromising their original physiological activities has become one of the relevant topics in flavonoid research [10,11,12].

Numerous studies have shown that the oligoglycoside moiety added to QU via enzymatic modification might increase the hydrophilic properties and enhance the bioavailability of the obtained substance. Such a product dissolves easily in the gastrointestinal fluids and is readily digested at the intestinal mucosa membranes to yield QU ready for absorption. Enzymatically modified isoquercitrin (EMIQ) is one such innovative substance. It is a water-soluble quercetin oligoglucoside produced from RT by a series of enzymatic transformations. The bioavailability of EMIQ is approximately 17-times that of QU and 44-times that of RT; this molecule is also more easily absorbed in humans [10,13].

In Japan, EMIQ has already been approved as a nutraceutical and food additive in various beverages and foods [14], and the U.S. Food and Drug Administration (FDA) concluded that EMIQ is generally regarded as safe (GRAS Notice 220) for use as an antioxidant [15]. Furthermore, over the past two decades, several studies have been conducted to verify the bioavailability, pharmacological activity, and safety of EMIQ, which define its therapeutic value in more detail. The available data suggest that EMIQ may be a promising therapeutic substance with broad biological potential, especially as an anti-allergic [16,17,18,19], cardioprotective [20,21,22], and chemopreventive agent [23,24,25,26,27,28,29,30,31,32].

Despite the advance that has been made in research on the different pharmacological properties of EMIQ, no comprehensive reviews are available. Thus, this paper summarizes the findings on the production methods, physicochemical properties, biological effects, and therapeutic potential of this molecule.

## 2. Structure, Production, and Properties

### 2.1. Structure

**Figure 1:**
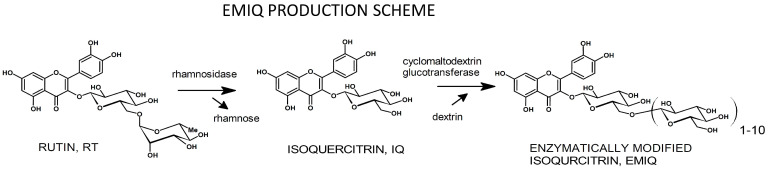
A simplified scheme for EMIQ production. RT obtained from natural sources is transformed using bacterial enzymes first into IQ and then into isoquercitrin oligoglucosides with 1 to 10 α-glucosyl moieties. For more details, see the manuscript text.

EMIQ, also known as AGIQ, is an α-glycosyl isoquercitrin (α-oligoglucosyl quercetin 3-*O*-glucoside) produced by San-Ei Gen F.F.I., Inc. (Toyonaka, Japan) through enzymatic conversion of RT into a mixture of IQ and its α-glucosyl derivatives with 1–10 α-glucose moieties connected linearly via 1→4 linkage (IQG_1_-IQG_10_) (Figure 1). The mean content of IQ-IQG_7_ in the mixture is over 94%; free QU is present at less than 1%. The average molecular weight of EMIQ is about 800 Daltons [15,33].

### 2.2. Production

RT, required for EMIQ production (Figure 1), is isolated from the buds or flowers of *Styphnolobium japonicum* (L.) Schott., the entire plant of *Vigna angularis* (Willd.) Ohwi & H. Ohashi, or the entire plant of *Fagopyrum esculentum* Moench [14,15,34].

In the first stage of the production process, α-L-rhamnosidase (from *Penicillium decumbens*; EC. 3.2.1.40) leads to the derhamnosylation of RT to produce IQ. Next, an aqueous solution of IQ is combined with dextrin and treated with cyclomaltodextrin glucanotransferase (from *Paenibacillus pseudalcaliphilus* or *Paenibacillus macerans*; EC.2.4.1.19), which catalyzes transglycosidation and incorporates α-glucose moieties to form EMIQ. The solution is heated (98 °C for 5 min at pH 4.2) to inactivate both enzymes, then filtered and evaporated under vacuum. The residue is dissolved in ethanol and subjected to crystallization, which removes impurities, including QU. The final ethanolic solution is evaporated in vacuo yielding EMIQ [15]. All enzymes used in the production of EMIQ are on the List of Existing Food Additives in Japan’s Food Sanitation Law and are produced under food additive good manufacturing practices (GMPs) in Japan [15,35].

### 2.3. Physicochemical Properties

EMIQ is a yellow/orange/yellow-brown powder with a distinctive odor [14,15]. The octanol-water partition ratio (logP = −0.77) expressing the lipophilicity of a compound shows that the EMIQ has substantial hydrophilic properties [13]. The solubility of EMIQ in distilled deionized water at 25 °C is 130 g/L, which is ~81,500- and ~8400-times higher than observed for QU (0.0016 g/L) and RT (0.0155 g/L), respectively [9]. Moreover, EMIQ has a very high resistance to oxidative degradation and higher thermal stability than QU. It completely resisted the oxidation process in the presence of Cu^2+^ ions (0.1 mM, pH 3.5) for up to 14 h at 25 °C, whereas QU was degraded entirely under the same conditions. The change in reaction conditions, i.e., an increase in the concentration of Cu^2+^ ions, alkalinity, and temperature, caused EMIQ to decompose faster, but its retention was still higher than that of QU. During thermal exposition at 100 °C for 3 h, the amount of EMIQ was reduced by 55%, while QU was undetectable by HPLC after 3 h at 80 °C. On the other hand, oligoglucosylation does not protect flavonoid aglycones from degradation by irradiation with ultraviolet C light (UV-C) at 254 nm [9].

## 3. Application in the Food Industry

EMIQ was first developed in 1987 and approved by the Japanese Ministry of Health and Welfare for use as a food additive in 1996 [36]. Its monograph with quality requirements is contained in the latest edition of Japan’s Specifications and Standards for Food Additives [14]. The EMIQ producer distributes it under the commercial names SANMELIN^®^ AO-3000 and SANMELIN^®^ POWDER R-20 and recommends EMIQ as an antioxidant for preserving flavor, color, and freshness [34].

In the USA, the Expert Panel of the Flavor and Extract Manufacturers Association concluded that EMIQ might be generally recognized as safe (GRAS) in 2005 [36]. The U.S. FDA also granted GRAS status for EMIQ. Currently, in the United States, EMIQ is approved as an antioxidant (for protection of flavors and colors) in multiple food categories (i.e., fruit and vegetable juices, beverages, milk products, puddings, jams, jellies, bakery products) at 75–150 milligrams per kilogram food (mg/kg) and in chewing gum at 1500 mg/kg [15].

The usability of EMIQ as a color protector in beverages was demonstrated, e.g., by Yan et al. [37], who proved that EMIQ remarkably improves the thermal and light stability of anthocyanins at different pH levels. It was also shown to prevent, to some extent, the lipid peroxidation of a walnut beverage emulsion induced by thermal treatment and UV light. However, in that case, its activity was worse than that of a standard synthetic antioxidant, BHT. Moreover, the emulsion system with EMIQ was prone to the loss of its stability [38]. Thus, as a strongly hydrophilic molecule, EMIQ might not be particularly suited for use in lipid emulsions.

## 4. Metabolism, Absorption, and Bioavailability

### 4.1. Upper Gastrointestinal Tract

**Figure 2:**
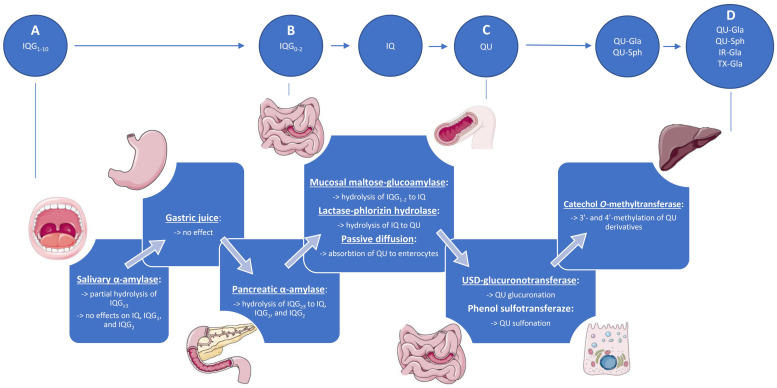
Metabolic pathway of EMIQ after oral ingestion. (**A**) Oral ingestion of EMIQ in the form of isoquercitrin oligoglucosides with 1 to 10 α-glucosyl moieties; (**B**) after passing the upper gastrointestinal system, the α-oligoglucosyl chain of EMIQ components is shortened to at most two moieties due to the activity of salivary and pancreatic amylases; (**C**) the enzymes of the intestinal brush border free QU from the glycosides with IQ as an intermediate product; QU is then absorbed into the enterocytes via passive diffusion; (**D**) the metabolites of EMIQ detectable in the plasma are formed in the enterocytes and in the liver by coupling QU with glucuronic acid (Gla), sulphates (Sph), or by methylation of QU to form isorhamnetin (IR) and tamarixetin (TX).

The metabolic pathway of EMIQ after oral ingestion (Figure 2) starts already in the mouth. In vitro assays showed that salivary α-amylase is able to cleavage parts of the α-(1→4) linkages and deoligomerize the EMIQ components into simpler ones. The conversion is the faster the longer the α-glucosyl side chain is. Even after 1 min of contact, substantial degradation was observed, especially for IQG_6_ and IQG_7_, of which only about 20% or less was left intact. On the other hand, IQ, IQG_1_, and IQG_2_ were resistant to the α-amylase digestion even after a longer exposure time [39].

The experiments with artificial gastric juice showed that all EMIQ components are resistant to the low pH of the stomach for up to 3 h at 37 °C, which suggests that no substantial digestion processes take place in this part of the gastrointestinal system [39]. Further degradation of EMIQ occurs in the duodenum and is due to the action of pancreatic α-amylase. In comparison to its salivary counterpart, some variations of the enzyme reactivity were noticed that resulted in different product distributions. Nevertheless, the enzymatic cleavage was still hardly effective towards IQ, IQG_1_, and IQG_2_, which constitute the final digestion products at this stage. It was also established that IQG_3_ and IQG_4_ degrade mainly into IQ and IQG_1_, while IQG_2_ is the dominant product of IQG_5_ and IQG_6_ transformation. The overall results of the study suggest that, upon arriving in the small intestine, most of the EMIQ might be already converted into IQ, IQG_1_, and IQG_2_ [39].

### 4.2. Lower Gastrointestinal Tract

In the small intestine, the remaining α-glucosyl derivatives might be degraded into QU in a two-stage process, with IQ as an intermediate product. The in vitro study with the homogenate of rat intestinal epithelium showed that the process goes rapidly, and the inhibition of α-glucosidase activity hindered the first phase of the transformation. Thus, mucosal maltose-glucoamylase (MGAM) was suggested to be involved [10,18]. MGAM is one of the α-glucosidases of the brush border epithelial cells that takes part in the final stage of starch decomposition. It cleaves glucose units from the non-reducing end of the sugar chain by hydrolysis of α-(1→4)-glucosidic linkages [40]. Thus, it might be at least partially responsible for the observed conversion. However, more detailed experiments with EMIQ components are still required to explain the role of MGAM (or other α-glucosidases) at this stage of their digestion.

The transformation of IQ into QU is a better-recognized process. In the experiments of Makino et al. [10,18], lactase-phlorizin hydrolase (LPH) was found to be the primary enzyme responsible for the conversion. LPH is a membrane-bound member of the β-glycosidase family present in the mammalian intestine. It is the only β-glycosidase that acts extracellularly; thus, it can operate on substrates prior to the absorption. It is known to be responsible for the hydrolysis of various dietary flavonoids, including IQ (K_m_ = 46 µM) [41]. As phlorizin did not influence IQ hydrolysis, it was suggested that the lactase domain of LPH participates in the process [41,42]. The glucose released during the reaction is actively transported into the enterocytes by a sodium-dependent glucose transporter (SGLT 1), while the aglycone is now lipophilic enough to be absorbed via passive diffusion [18,41].

As the metabolites of EMIQ are present in the blood as early as 15 min after oral administration, most researchers indicated the small intestine as the leading absorption site [10,18]. It is, however, known that some amounts of the polyphenols may reach the large intestine, where they undergo transformation by gut microbiota. Among the bacterial catabolites of QU are, e.g., 3,4-dihydroxyphenylacetic acid, 3-hydroxyphenyl acetic acid, and 3-(3-hydroxyphenyl)-propionic acid [43,44]. However, none of the studies on EMIQ’s bioavailability have looked at its fate beyond the small intestine, and the plasma concentrations of the products of bacterial QU degradation have yet to be measured. Thus, this topic is undoubtedly worthy of further investigation.

### 4.3. Plasma Metabolites and Bioavailability 

**Figure 3:**
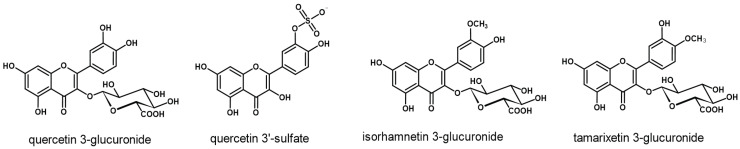
Some of the primary metabolites of EMIQ after oral administration.

The primary metabolites of EMIQ thus far detected in plasma after oral administration to animals and humans are QU conjugates. Some methylated derivatives have also been detected, in particular the conjugates of isorhamnetin and tamarixetin (Figure 3, Table 1) [10,13,45,46]. The transformation of QU to glucuronides and sulfates mostly takes place in the enterocytes due to the action of UDP-glucuronosyltransferase and phenol sulfotransferase, respectively [42,44]. On the other hand, catechol *O*-methyltransferase, responsible for the methylation process, is present in larger quantities in the liver than in the intestine [10]; thus, at least to some extent, this part of the metabolic transformation may occur after the absorption of QU conjugates to the blood. Following this hypothesis, the plasma levels of methylated metabolites remain at a plateau for a long time (in contrast to the levels of QU conjugates, which gradually decrease after the initial rise) [10,13].

Animal research showed that the true bioavailability (AUC_oral_/AUC_intravenous_ (%)) of EMIQ after oral administration, assessed as total plasma QU levels, was 35%. Thus, it was about 17-, 3-, and 44-times higher than that of QU (2%), IQ (12%), and RT (0.8%), respectively [10].

In the only study with human participants, quercetin 3-glucuronide, quercetin 3ʹ-sulphate, and isorhamnetin 3-glucuronide have been reported as the main metabolites. A substantial variation was observed between individual patients in terms of the quantitative ratios between different metabolites. However, in general, the metabolite profile of EMIQ was similar to that after IQ administration. The maximal plasma concentration of the metabolites was achieved after 1.5–2 h and, similar to the case of animal studies, was about three-times higher than that obtained for IQ [13].

### 4.4. Elimination and Accumulation

EMIQ’s metabolites are at least partially eliminated through urine, and the elevated levels of QU conjugates in the urine samples of laboratory animals were detected even 28 days after exposure. This fact suggests that the metabolites may to some extent accumulate in the body. Indeed, the quantifiable amounts of quercetin 3-glucuronide, isorhamnetin, QU, and kaempferol were detected in the bones, cerebrum, and fat of Sprague-Dawley rats after 14 days of oral administration of EMIQ (1.5, 3, and 5% in the diet). The accumulation was dose-dependent and caused the characteristic yellow discoloration of the femur. The data suggest that the effect is reversible and not connected with any histopathological changes. On the other hand, this is evidence of the systemic availability of EMIQ metabolites, which might be advantageous for its health benefits [36].

## 5. Safety

The so-far collected data on EMIQ toxicity, performed on animal models, indicate that EMIQ is safe, does not induce severe adverse effects, and does not exhibit cytotoxic, genotoxic, embryotoxic, or allergenic effects (Table 2).

### 5.1. General Toxicity

The acute toxicity assessment of EMIQ was commissioned by San-Ei Gen F.F.I. for the GRAS notification [15]. The rats were treated with a single dose of EMIQ up to 25 mg/kg and then observed for 14 days for adverse reactions. No deaths were recorded during the follow-up period; thus, the LD_50_ for rats was estimated to be greater than 25 mg/kg. Moreover, no abnormal behavior or other toxic effects were documented, and the necropsy found no significant abnormalities.

The toxicity tests with prolonged EMIQ exposure in rat/mouse models were carried out within 4–104 weeks and used EMIQ concentration in the diet up to 5%. No treatment-related mortality or severe clinical symptoms was reported in any of the studies [15,45,47,48,49]. In animals consuming higher concentrations of EMIQ (≥2.5%), a tendency for lower body weight was noticed that mostly was unrelated to lower food consumption [45,47,49]. Dose-dependent yellow coloration of bones (e.g., femur, calvarium, cranium) was reported in every study; however, it was not connected with any histopathological changes in the bone structure [15,45,47,49]. Increased urine ketone level (only in males on 2.5% EMIQ dose) and unnaturally yellow urine coloration was observed by Tamano et al. [47], but generally, the urinalysis did not present any treatment-related changes of toxicological importance [45,48]. Statistically significant, but mild changes in the weights of different organs were reported in almost every study. They were not connected with any pathological findings and occurred only in one of the genders, and no clear trend seemed to be observable across the studies [45,47,48,49]. In a study by Nyska et al., no changes in automated motor activity were found, which suggest no neurotoxic effects [45]. Sporadic non-dose-dependent changes in hematological and biochemical parameters were found by Tamano et al. [47], Nyska et al. [45], Salim et al. [48], and Mahapatra et al. [49], which were considered of no toxicological meaning.

The subacute toxicity was also evaluated in a model of juvenile Göttingen minipigs. No toxic effects were found during a 10-day treatment with EMIQ in doses up to 1000 mg/kg/day. Compared to untreated control animals, there were no effects on weight gains and food consumption and no significant differences in hematological parameters and organ weights. The histological examination did not reveal any pathologies that could be related to the EMIQ-administration [46]. No severe adverse effects were found during a 4-week treatment with the same doses of EMIQ. All pigs fed with 1000 mg and two males treated with 300 mg of EMIQ had yellow discoloration of the femur and coccyx. However, microscopic observations presented no change in bone growth or histological changes. The consumption of EMIQ also affected the color of urine, without affecting any of the parameters of the urinalysis [46].

In the studies with human subjects, EMIQ (42 mg/day/6-times a week for 4 months) was well tolerated and no adverse effects were reported. The hematological and biochemical parameters of serum stayed within the reference ranges during the treatment time, and no significant changes connected with EMIQ supplementation were observed [50].

### 5.2. Genotoxicity

A comprehensive assessment of the genotoxic potential of EMIQ and IQ was conducted following the current EFSA (European Food Safety Authority), OECD (Organisation for Economic Co-operation and Development), and FDA guidance on genotoxicity and toxicity testing. Both substances (in doses up to 5000 μg/plate) tested positive in *Salmonella typhimurium* strains (TA98, TA100, TA1537) reverse mutation assays, and the exposure to IQ induced moderate chromosomal aberrations in Chinese hamster ovary cells. However, all other in vitro micronuclei and chromosomal aberration assays in mammalian cells gave negative results. Moreover, no in vivo genotoxicity was demonstrated in a micronuclei/comet assay in rats and a Muta^TM^ mouse mutation assay, thus supporting the safety of EMIQ usage [51].

**Table 2:** Toxicity studies of EMIQ.

Model	Sample Size	Dosage	Treatment Time	Effects	Reference
*Acute toxicity*					
Four-week-old male and female Sprague-Dawley rats	10/group/sex	16, 20, 25 mg/kg p.o.	Single dose + 14-day follow up	No mortality related to the treatment No toxic effects related to the treatment	[15]
*Subacute toxicity*					
Juvenile Göttingen minipigs	5/group	100, 300, 1000 mg/kg/day p.o.	10 days	No toxic effects related to the treatment	[46]
	7/group	100, 300, 1000 mg/kg/day p.o.	4 weeks	No clinical signs nor mortality related to the treatment Tendency for lower body weight gain Darker urine Yellow coloration of bones (femur and calvarium)	
4-week-old male and female F344/DuCrj rats	5/group/sex	0.625, 1.25, 2.5, 5% in the diet	4 weeks	No clinical signs nor mortality related to the treatment Yellow coloration of bones (cranium, forelimb, and hindlimb bone) Tendency for lower body weight (>2.5%) Tendency for lower food and water consumption (>2.5%, males) No impact on organ weights	[15]
*Subchronic toxicity*					
4-week-old male and female F344 rats	10/group/sex	0.3, 0.625, 1.25, 2.5% in the diet	13 weeks	No clinical signs nor mortality related to the treatment Tendency for lower body weight (2.5%, females) No impact on food and water consumption Yellow coloration of the urine Higher ketones level in the urine (2.5%, males) Yellow coloration of bones (femur and cranium) Higher reticulocytes count Increased serum levels of γ-GTP and BUN Mild changes in various organ weights without morphological alterations	[47]
5-week-old male and female Sprague-Dawley rats	10/group/sex	0.5, 1.5, 3, 5% in the diet	90 days	No clinical signs nor mortality related to the treatment No impact on body weight No impact on food consumption Yellow coloration of bones (femur, calvarium, maxilla) Mild changes in various organ weights without microscopic abnormalities No changes in urinalysis No changes in automated motor activity	[45]
*Chronic toxicity/Carcinogenicity*					
5-week-old male and female F344/DuCrj rats	50/group	0.5, 1.5% in the diet	104 weeks	No clinical signs nor mortality related to treatment No impact on body weight No impact on food and water consumption Decreased serum levels of γ-GTP Changes in various organ weight unrelated to neoplasia No evidence of carcinogenicity	[48]
5-week-old male and female rasH2 mice	25/group/sex	1.5, 3, 5% in the diet	6 months	No clinical signs nor mortality related to the treatment Lower body weight (females, 3%) Higher food consumption (males, 5%) Mild changes in various organ weights without pathologic findings Sporadic non-dose dependent hematological changes Yellow coloration of bones (femur, calvarium) No changes in urinalysis related to the treatment No evidence of carcinogenicity	[49]
*Maternal toxicity and embryotoxicity*					
New Zealand white female rabbits	22/group	250, 500, 1000 mg/kg/day p.o.	6–28 gestation day	No maternal toxicity No embryotoxic effects No teratogenic effects No effect on reproductive parameters	[52]
	48/group	500, 1000 mg/kg/day p.o.			
*Genotoxicity*					
Male and female B6C3F1 mice Male and female Sprague Dawley rats	5/group	1000, 1500, 2000 mg/kg/day p.o.	3 days	No clinical signs related to the treatment Slight mass loss in male animals (−3.3%) No biologically relevant increase in the frequency of MN-RET No biologically relevant increase in DNA damage in liver, duodenum, and stomach tissue	[51]
Male transgenic mice (Muta^TM^ Mouse)	5/group	5%, 1.5%, 0.5% in diet	28 days	Unnaturally yellow urine No other changes in clinical signs No impact on body weight, food consumption No differences in the organ weights and relative organ weights	[51]
*Allergenic effects*					
7-week-old female CBA/J mice	5/group	10%, 25%, 50% in DMF topically	3 days	No abnormal clinical observations, no mortality No significant dermal irritation symptoms No impact on the size of lymph nodes No sensitizing effects found by Local Lymph Node assay	[53]

### 5.3. Embryotoxicity

EMIQ was evaluated for embryo/fetal survival, developmental toxicity, and maternal side effects in female rabbits. No maternal toxicity was observed after oral administration of EMIQ in doses up to 1000 mg/kg/day. The reproductive parameters, such as gravid uterine weight, number of implantations, implantation loss, live young, the ratio of males-to-females, placental, litter and fetal weights, and litter size, were not affected. In fetal development, sporadic cases of kidney and ureter absence were recorded; however, they were considered unrelated to the treatment due to the lack of reproducibility. Based on the study results, the NOAEL for maternal toxicity and embryo/fetal development for EMIQ was specified as 1000 mg/kg/day [52].

### 5.4. Allergenic Effects

EMIQ exhibited no skin irritant properties when applied topically to murine ear in concentrations up to 50%. The compound did not induce lymphadenopathy, ear swelling, erythema, irritation, and other skin lesions. The skin sensitization potential of EMIQ was assessed by the Local Lymph Node Assay ex vivo, and according to the assay protocol, EMIQ has been classified as a non-sensitizer at all tested concentrations [53].

## 6. Bioactivity

The animal studies conducted so far showed that EMIQ administered orally can exhibit significant systemic effects (Table 3). Within the research interest were, e.g., anti-allergic, cardioprotective, and chemopreventive effects, which to a large extent are connected with the antioxidant and anti-inflammatory potential of EMIQ [19,20,24]. As the metabolite profile of EMIQ is similar to that of natural quercetin glycosides, it is often regarded as the more advantageous source of this multi-functional flavonol [20,54].

### 6.1. Cardioprotective and Metabolic Effects

In a study carried out on rats with spontaneous hypertension, it was found that EMIQ (3 and 26 mg/kg/day) has a significant hypotensive effect. EMIQ reduced systolic blood pressure in the tested rats by about 16–21 mmHg, but did not impact the diastolic blood pressure and heart rate. The EMIQ effects were higher than those of QU, but weaker than those of diltiazem (a positive control) [20].

The atheroprotective and plaque-stabilizing effects of EMIQ were examined using apolipoprotein E-deficient mice, which spontaneously develop hypercholesterolemia and atherosclerosis when fed a high-fat diet. After 14 days of therapy, EMIQ (0.026% in the diet) significantly reduced the atherosclerotic aortic lesions and the plaque area in the aortic sinus compared to the untreated group. In addition, EMIQ decreased the accumulation of macrophages (by about 47%), increased collagen content in the plaque (by about 41%), and almost doubled the number of smooth muscle cells, suggesting a plaque-stabilizing effect. However, the reduction of atherosclerosis was not connected with normalizing the lipid metabolism, as no impact on total cholesterol, high-density cholesterol, and triacylglycerol levels was revealed. The authors connected the beneficial effects of EMIQ with its antioxidant capacity and hindering lipid peroxidation. Moreover, they suggested that EMIQ acts directly in the arterial wall, which was evidenced by the decrease (about 47%) in the levels of 4-hydroxy-2-nonenal (a major aldehyde arising due to lipid peroxidation) in atherosclerotic lesions. On the other hand, the levels of TBARS in the serum were not affected [21].

Contrary effects as to the impact of EMIQ on lipid metabolism were found by Jiang et al. In their study, the authors compared the effect of QU and its glycosides (IQ, RT, and EMIQ) on the prevention of hyperglycemia and obesity in mice on a high-fat diet. All tested compounds reduced the amount of white adipose tissue in the mesentery. Moreover, supplementation with QU and its glycosides normalized the glycemia parameters, reduced the weight gain, lowered plasma lipid levels, and decreased markers of adipocyte differentiation in adipose tissue. According to the authors, the observed effects result from the increased phosphorylation of AMP-activated protein kinase (AMPK) and subsequent inhibition of cholesterol and triglyceride synthesis, suppression of adipocyte differentiation and lipogenesis, and activation of lipolysis. Furthermore, the investigated analytes induced the translocation of the muscle glucose transporter 4 (GLUT4), which plays a vital role in regulating whole-body glucose homeostasis [54].

In a recent study by Im et al. [55] in mice also, a synergistic effect of EMIQ in combination with a heat-transformed green tea extract (100 + 100 mg/kg bw) was observed in the reduction of body weight, fat mass, and adipocyte size. The therapeutic effects were comparable to that of mirabegron (10 mg/kg bw), an agonist of the β3-AR signaling pathway, the primary regulatory mechanism involved in lipolysis. Furthermore, in in vitro models of C3H10T1/2 adipocytes and immortalized brown pre-adipocytes, it was proven that co-treatment (50 + 50 mg/kg bw) contributes to an increase in mitochondrial metabolism and a decrease in lipid content in adipocytes.

On the other hand, the consumption of EMIQ (7 g/kg bw) in combination with soybean fiber (50 g/kg bw) ameliorated glucose intolerance induced by a high-fat high-sucrose diet in rats. The effect was ascribed to EMIQ metabolites, which were suggested to increase the secretion of GLP-1 [56]—an incretin that regulates the postprandial increase in glucose by several mechanisms, including promoting insulin gene transcription, stimulating pancreatic β cell proliferation and neogenesis, inhibiting β cell apoptosis, and blocking glucagon release [57]. However, most observed effects were not significant in the groups administered single treatments. Such results were explained by the increased bioavailability of QU from EMIQ during co-ingestion of soybean fiber, which was confirmed by the increased levels of its conjugates in blood. As the soybean fiber mainly impacts the fermentation in the large intestine, the elevated absorption of QU may result from changes in the growth and behavior of intestinal microflora [56].

### 6.2. Anti-Allergic and Anti-Inflammatory Effects

The studies of Makino et al. [18] showed that EMIQ might be effective in hindering allergic reactions. Its activity (single oral dose of 4 mmol/kg) in inhibiting passive cutaneous anaphylaxis in mice sensitized by intraperitoneal ovalbumin injection was comparable to that of azelastine (3.3 µmol/kg). EMIQ treatment (50 and 100 mg/kg) also effectively reduced mice paw edema induced by histamine and compound 48/80. The higher dose of EMIQ was as efficient as sulfasalazine (100 mg/kg) and, similar to the control drug, completely prevented fatal anaphylactic reactions. In addition, the histopathological data revealed that EMIQ prevented mast cell degranulation and reduced histamine release [19].

In two small (20–24 participants) double-blind, placebo-controlled clinical trials, the potential of EMIQ to alleviate pollinosis symptoms was evaluated. In both cases, during 8-week treatments with EMIQ (100 mg/day), a clear trend was noticed toward reducing ocular symptoms such as itching, lacrimation, and congestion. While the decreases in the scores for individual symptoms were mainly not statistically significant, a significant reduction in the total scores for ocular symptoms was recorded. On the other hand, no differences were found in the nasal symptoms. In one of the studies, after a 4-week treatment, an improvement was noticed regarding the impact of the symptoms on daily activities; however, the overall quality of life was not affected in both trials. Moreover, there were no changes in the plasma IgE, IL-4, IL-5, IL-12, IL-13, IFN-γ, and eotaxin levels. Nevertheless, the potential of EMIQ to alleviate allergic reactions in human subjects is undoubtedly worthy of further investigation [16,17].

The anti-inflammatory properties of EMIQ have also been studied in the context of digestive system disorders. For example, EMIQ (50 and 100 mg/kg, orally) demonstrated significant protective effects in mice with gastric ulcers induced by water-restraint stress. At the higher dose, no major gastric mucosa lesions were found, and only a slight hemorrhage and a few exposed superficial mucus cells were observed. The authors related the results with the decrease in histamine release and prevention of its ulcerative impact on the stomach. The gastroprotective effect of EMIQ may also have antioxidant mechanisms. In particular, a dose-dependent increase of gastric reduced glutathione levels and a reduction in gastric lipid peroxidation markers (malondialdehyde) and inflammation mediator (nitric oxide) were noticed [19].

EMIQ treatment (1.5% in diet) also helped to counter the adverse inflammatory responses in dextran-sodium-sulfate-induced colorectal mucosal injury in mice by reducing the expression of pro-inflammatory mediators, especially TNF-α, IL-6, IL-17, and a keratinocyte-derived cytokine. Histopathological findings showed that the animals treated with EMIQ had milder submucosal edema and colonic mucosa damage than those not taking the drug [31,58]. Microarray gene expression analysis showed that the anti-inflammatory effects could result from the regulation of the various signaling pathways, i.e., mitogen-activated protein kinase (MAPK), transforming growth factor β (TGF β), IFN-γ, or focal adhesion [58].

### 6.3. Chemopreventive Effects

Yokohira et al. [32] were the first to investigate the chemopreventive effects of EMIQ. The study was motivated by the high antioxidant properties of the substance and its ability to increase the antioxidant capacity of rat plasma when administered with the diet. In the rat model of liver carcinogenesis induced by *N*-diethylnitrosamine (DEN), a correlation was found between EMIQ dose (0.01, 0.1, and 1% in the diet) and the decrease in the number of preneoplastic lesions (glutathione-S-transferase-placental-form (GST-P)-positive foci). However, the individual inter-group differences were not statistically significant. As one of the possible reasons for the lack of significance, a generally poor response to the carcinogenic effect of DEN was given.

In the following years, a series of experiments was performed using a two-stage hepatocarcinogenesis model, in which the administration of other tumor promoters followed DEN treatment. In all of the studies, EMIQ treatment (0.2% in drinking water or 0.5% in the diet) significantly decreased the number and area of GST-P-positive foci in the liver [23,24,26,27,29]. The effects of EMIQ were also expressed by diminishing the number of proliferating cells and increasing the number of apoptotic cells inside the lesions [26,27,29]. The studies were, however, inconclusive as to the potential mechanisms behind such results.

In the study of Nishimura et al. [23], in which the carcinogenesis was induced by co-treatment with DEN and oxfendazole, EMIQ administration reduced the transcription of the *Cyp2b2* gene, which increased due to the carcinogens exposure. Cytochrome P450 2B2 (CYP2B2), coded by that gene, is one of the enzymes of phase I drug metabolism and might be involved in the generation of oxidative stress, which, in turn, might be responsible for the formation of preneoplastic lesions. Thus, the antioxidant properties of EMIQ were considered responsible for its chemopreventive activity. An in vitro study in which EMIQ hindered the NADPH-dependent ROS production in hepatic microsomes further supports this hypothesis. Moreover, in the study by Hara et al. [24], EMIQ suppressed the lipid peroxidation induced by DEN/piperonyl butoxide treatment, which was evidenced by the decrease in the TBARS level in rat liver [29]. The antioxidant mechanism was also implicated in EMIQ’s prevention of cancer development induced by DEN/β-naphthoflavone. In this case, the impacted genes were *Gstm1* and *Yc2*, involved in phase II drug metabolism and protection against oxidative stress.

On the other hand, Morita et al. [26] did not confirm the antioxidant effects of EMIQ in the DEN/phenobarbital-induced model of liver cancer. In the study, there were no differences between the levels of ROS and oxidative stress markers (TBARS and 8-OHdG) in the livers of rats treated with EMIQ and untreated control. No significant changes were recorded for the mRNA expression levels of the genes coding enzymes of the cytochrome P450 family, including CYP2B2. As the primary mechanism of the chemopreventive effects in that model, the inhibition of nuclear translocation of constitutive androstane receptor (CAR) was suggested. CAR is one of the xenobiotic receptors regulating the expression of numerous drug-metabolizing enzymes and is known to be responsible for the carcinogenic effects of phenobarbital and other non-genotoxic chemicals in rodents [59,60]. However, the exact way EMIQ interferes with the process still needs to be explained.

**Table 3:** Bioactivity studies of EMIQ.

Animal	Model	Treatments	Sample Size	Effects of EMIQ Treatment	Reference
*Cardioprotective and metabolic effects*					
4-week-old male SHR/Izm rats	Spontaneous hypertension	EMIQ (3, 26 mg/kg p.o.), QU (1.2, 10.4 mg/kg p.o.), diltiazem (120 mg/kg p.o.)	10/group	↓ systolic blood pressure No effect on diastolic blood pressure No effect on heart rate	[20]
6-week-old male apoE-deficient mice	Diet-induced atherosclerosis	EMIQ (0.026% in the diet)	7–10/group	No effect of food intake, body weight, and lipid profile ↓ (~50%) area of aortic atherosclerotic lesions ↓ (~24%) plaque area in the aortic sinus ↓ lipid peroxidation product (4-HNE) in the plaque area ↓ macrophage accumulation in the plaque ↑ plaque stabilization (↑ collagen levels and smooth muscle cell accumulation)	[21]
4-week-old male ICR mice	Diet-induced obesity and hyperglycemia	EMIQ, QU, IQ, RT (0.1% in the diet)	5/group	↓ weight gain ↓ glucose, insulin, ↓ HOMA-IR ↓ total cholesterol, triglycerides, NEFA ↑ AMPK phosphorylation, no effect on AMPK expression ↑ GLUT4 translocation to the plasma membrane of skeletal muscle No effect on JAK/STAT-pathways ↑ ACC phosphorylation in white adipose tissue and the liver ↓ adipocyte differentiation ↓ UCP1, PGC-1α, PRDM 16 expression in white adipose tissue ↓ FAS, SREBP1, ↑ CPT1, PPARα expression in the liver	[54]
5-week-old male Wistar-ST rats	Diet-induced obesity and hyperglycemia	EMIQ (0.7% in the diet) Soybean fiber (5% in the diet) EMIQ + soybean fiber (0.7% + 5% in the diet)	7/group	↓ visceral fat (soybean fiber) ↓ glucose, insulin, HOMA-IR (EMIQ + soybean fiber)	[56]
6-week-old male C57BL/6 mice	Diet-induced obesity	EMIQ + heat transformed green tea (50 + 50 mg/kg, 100 + 100 mg/kg p.o.) Mirabegron (10 mg/kg p.o.)	6/group	↓ body weight and fat mass ↓ adipocyte size No effect on fecal fat content ↑ VO_2_, VCO_2_, and energy expenditure ↑ brown adipocyte markers and mitochondrial proteins (UCP1, COXIV) ↑ MCAD in brown and white adipose tissue ↑ glucose tolerance	[55]
*Anti-inflammatory and anti-allergic effects*					
6-week-old male Balb/c mice	Sensitization with ovalbumin	EMIQ	7–8/group	↓ passive cutaneous anaphylaxis (4 mmol/kg)	[18]
8-week-old male Swiss albino mice	Histamine-induced paw edema	EMIQ (50, 100, 200 mg/kg p.o.); sulfasalazine (100 mg/kg p.o.)	5/group	↓ paw edema (at 50, 100 mg/kg) comparable to SSZ ↓ paw edema (at 200 mg/kg) better than SSZ	
	Cpd 48/80-induced local paw edema	EMIQ (50, 100 mg/kg p.o.); sulfasalazine (100 mg/kg p.o.)	5/group	↓ paw edema (at 50, 100 mg/kg) comparable to SSZ	[19]
	Cpd 48/80-induced systemic anaphylaxis	EMIQ (50, 100 mg/kg p.o.); sulfasalazine (100 mg/kg p.o.)	10/group	↑ survival to 100% (at 100 mg/kg) ↓ histamine release (at 50, 100 mg/kg) ↓ mast cells’ degranulation (at 50, 100 mg/kg)	
	WRS-induced acute gastric ulcer	EMIQ (50, 100 mg/kg p.o.); sulfasalazine (100 mg/kg p.o.)	5/group	Protection of gastric mucosa, ↓ hemorrhage, ↓ pathological changes, ↓ loss of superficial mucous cells ↑ reduced glutathione ↓ malondialdehyde, nitric oxide	
4-week-old female BALB/ cAnNCrlCrlj mice	Acute colitis induced by dextran sodium sulphate	EMIQ (1.5% in the diet)	4/group	↓ DSS-mediated decrease in BrdU-positive cell number ↓ colitis severity	[58]
4-week-old female BALB/ cAnNCrlCrlj mice	Acute colitis induced by dextran sodium sulphate	EMIQ (1.5% in the diet)	12/group	↓ diarrhea ↓ IL-6, TNF-α, keratinocyte-derived cytokine ↓ mucosal injury, ↑ mucinous production	[31]
*Chemopreventive effects*					
5-week-old male F344/NSlc rats	Liver cancer induced by N-diethylnitrosamine and oxfendazole	EMIQ (0.2% in drinking water) Melatonin (0.01% in drinking water)	5–12/group	↓ number of GST-P-positive foci ↓ transcription of *Cyp2b2* and *Me1*	[23]
6-week-old male F344/N rats	Liver cancer induced by N-diethylnitrosamine and β-naphtoflavone	EMIQ (0.2% in drinking water)	11–12/group	↓ area and number of GST-P-positive foci ↓ COX-2-positive cells ↓ mRNA expression of *Gstm1*, *Serpine1*, *Cox2* and *Nfkbia* ↑ mRNA expression of *Yc2*	[24]
4-week-old male F344/DuCrlCrlj rats	Liver cancer induced by N-diethylnitrosamine	EMIQ (1, 0.1, 0.01% in the diet) IQ (1, 0.1, 0.01% in diet) Purple corn color (1, 0.1, 0.01% in diet)	16–22/group	No effect on body weight and liver weight Negative dose–effect correlation for number of GST-P-positive foci ↑ antioxidant capacity of serum (at 1% EMIQ in the diet)	[32]
5-week-old male F344/N rats	Liver cancer induced by N-diethylnitrosamine and phenobarbital	EMIQ (0.2% in drinking water)	11–15/group	↓ area and number of GST-P-positive foci ↓ PCNA-positive (proliferating) liver cells No changes in ROS, TBARS, and 8-OHdG ↓ transcription of *Mapkapk3* and *Mrp2* ↓ nuclear translocation of CAR	[26]
5-week-old male F344/NSlc rats	Liver cancer induced by N-diethylnitrosamine and thioacetamide	EMIQ (0.5% in the diet)	11–12/group	↓ area and number of GST-P-positive foci ↓ PCNA-positive (proliferating) liver cells ↓ ED2-, COX-2-, and HO-1-positive hepatic macrophages ↓ CD3-positive lymphocytes ↑ TUNEL-, DR5-, and 4-HNE-positive liver cells inside GST-P-positive foci ↓ TUNEL- and DR5-positive liver cells outside GST-P-positive foci ↓ transcription for *Tnfrsf10b* No changes in the transcription level of antioxidant enzymes (*Aldh1a1*, *Gstm1*)	[27]
5-week-old male F344/NSlc rats	Liver cancer induced by N-diethylnitrosamine and piperonyl butoxide	EMIQ (0.2% in drinking water)	11–12/group	↓ area and number of GST-P-positive foci ↓ Ki-67-positive (proliferating) liver cells ↓ transcription for *Cyp1a1* (no statistical significance) ↑ transcription for *Mapk8*, *Mapk14*, and *Tp53* No impact on microsomal ROS production ↓ TBARS in the liver	[29]
5-week-old male F344/N rats	Liver cancer induced by N-diethylnitrosamine and malachite green + high-fat diet	EMIQ (0.5% in drinking water)	9–13/group	↑ liver weight ↓ total cholesterol and ALP No impact on aera and number of GST-P-positive foci No impact on proliferating liver cells (Ki-67-positive) ↓ p22phox-positive cells in GST-P-positive foci	[30]
4-week-old male F344/NSlc rats	Ochratoxin A-induced renal carcinogenesis	EMIQ (0.2% in drinking water)	15–16/group	No effect on the number of karyomegalic cells No effect on the number of immunoreactive cells for any marker ↓ transcription of *Mapk8* and *Txn1*	[61]
4-week-old female BALB/cAnNCrlCrlj mice	Inflammation associated colon carcinogenesis induced by azoxymethane and dextran sodium sulphate	EMIQ (1.5% in the diet)	8/group	↓ colon weight ↓ lesions with mucin-depleted foci and aberrant crypt foci ↓ multiplicity of the lesions ↑ β-catenin expression in the proliferating cell ↓ Iba1 and cyclin D1 scores	[58]
*Neurological effects*					
Mated female Slc:SD rats and their offspring	Developmental stage of rats	EMIQ (0.5% in the diet)	19/group	No impact on short-term spatial memory (Y-maze test) ↑ fear extinction learning (↓ freezing time in the 3rd trial) ↑ FOS-positive cells in the granule cell layer (hippocampus) ↑ transcription of *Chrm2*, *Slc17a6*, *Fos*, *Ntrk2*, and *Kif21b* in the hippocampal dentate gyrus ↑ transcription of *Grin2d* in amygdala ↑ transcription of *Chrna7*, *Kif21b* in infralimbic cortex	[62]
Mated female Slc:SD rats and their offspring	Developmental stage of rats	EMIQ (0.5% in the diet)	10/group	No differences in open-field test and object recognition test ↑ fear extinction learning (↓ freezing time) ↑ FOS- and p-ERK1/2-positive cells in the infralimbic cortex ↑ p-ERK1/2-positive cells in the prelimbic cortex Significant changes in genes transcription in hippocampal dentate gyrus, prelimbic and infralimbic cortex, and amygdala (Ephs/Ephrins, glutamate receptors, glutamate transporters, nitric oxide synthases, angiogenesis-related proteins, and others)	[63]
Mated female Slc:SD rats and their offspring	LPS-induced autism-like behaviors and disruptive hippocampal neurogenesis	EMIQ (0.25, 0.5% in the diet)	10/group	↑ moving distance in social interaction test during adolescent stage ↑ freezing time in contextual fear conditioning test ↓ number of GFAP-positive astrocytes ↑ recovery of mature granule cells Amelioration of fear memory acquisition ↓ neuroinflammation	[64]
*Musculotropic effects*					
6-week-old male ICR mice	Overload-induced hypertrophy initiated by ablation of the synergistic gastrocnemius and soleus muscles	EMIQ (4 mg/kg/day p.o.)	9/group	↑ cross-sectional area of the plantaris muscle ↑ minimal fiber diameter of the plantaris muscle	[65]
19-month old female C57BL/6J mice	Old age	EMIQ (0.3% in the diet)	7–9/group	No effect on muscles weight ↑ fat oxidation and energy consumption during exercise No effect on the glycolytic metabolites in the muscles ↑ antioxidant capacity of plasma ↑ transcription of *Gpx* ↓ carbonylated protein content in the muscles	[66]

The arrows ↑ and ↓ designate the increase or decrease in a particular parameter, respectively.

The role of the anti-inflammatory effects of EMIQ was indicated in the studies of Shimada et al. [24] and Fujii et al. [27]. The evidence was, among others, the decrease in the expression of genes connected with the inflammatory response, including *Cox2*, *Nfkbia*, and *Serpine1*, and the decrease in the number of COX-2-positive cells. Furthermore, according to Fujii et al. [27], the anti-cancer influence of EMIQ in DEN/thioacetamide-induced carcinogenesis also relies specifically on the suppression of the activation of ED2-positive hepatic macrophages.

Some evidence also suggested the impact of EMIQ on the mitogen-activated protein kinase (MAPK) cascade involved in cell growth. However, the collected data are ambiguous and indicated a rather indirect influence [26].

The latest study in a similar model of DEN/malachite-green-induced liver carcinogenesis showed no hindering impact of EMIQ on the development of neoplastic lesions [30]. However, in that case, the rats were additionally fed a high-fat diet to imitate steatosis-related liver promotion, which, combined with a different carcinogen used, might be a reason for the lack of EMIQ’s effectiveness. The concurrently tested apocynin, an inhibitor of NADPH oxidase (NOX), exhibited a chemopreventive effect, which was suggested to be connected with the reduced expression of one of the NOX membrane regulatory subunits (*p22phox*) in the lesions. A tendency to suppress *p22phox* expression was also observed for EMIQ, but the effect was not statistically significant. Nevertheless, EMIQ was able to diminish total cholesterol levels and the levels of alkaline phosphatase, one of the signs of liver dysfunction [30].

On the contrary, no chemopreventive effects of EMIQ were observed in the renal carcinogenesis model with karyomegaly induced in rats by administration of ochratoxin A. The authors suggest that the genotoxic effects of ochratoxin A is unrelated to oxidative stress, as the treatment did not impact the TBARS and reduced the glutathione levels [61]. This result might be indirect evidence for the role of antioxidant mechanisms in the chemopreventive properties of EMIQ.

Further confirmation of the role of anti-inflammatory mechanisms came in turn from the study of Kangawa et al. [58] in which EMIQ inhibited the colon carcinogenesis induced in mice by azoxymethane and dextran sodium sulfate treatment. The EMIQ administration reduced the number of proliferative lesions in vivo, hindered the macrophage infiltration in the intestinal wall, and decreased the histological inflammatory score. The impact on Wnt/β-catenin signaling was also implicated, but further studies are required to clarify the details.

### 6.4. Neurological Effects

Oxidative stress is also known to impact the neurological connections with the central nervous system and impair cognitive functions. Thus, some researchers decided to investigate the potential of EMIQ to mitigate those disorders.

Okada et al. [62] studied the impact of EMIQ on the synaptic plasticity in the hippocampus during the developmental stage in rats. The rats were treated with EMIQ (0.5% in the diet) starting from the pregnancy period up to adulthood. No impact of the treatment on short-term spatial memory was found, as judged by the results of the Y-maze test. However, the fear extinction tests revealed a decrease in the freezing time in the third trial, thus suggesting that EMIQ might support fear extinction learning. Moreover, the increase in synaptic plasticity was indicated by induced transcription of some proteins associated with long-term memory and memory rewriting (e.g., *Fos*, *Kif21b*, *Grin2d*) in the hippocampal dentate gyrus, amygdala, and prefrontal cortex. In the subsequent investigation, it was determined that EMIQ exposure starting already in the fetal stage is required to obtain the advantageous effects. Thus, some EMIQ-induced changes occur very early in brain development. Additionally, more detailed immunohistochemical assays revealed a much more complex impact on the proteins involved in synaptic plasticity [63].

In the study by Okano et al. [64] in a rat model, it was also demonstrated that EMIQ treatment (0.25 and 0.5% in the diet), initiated in late pregnancy and continued into adulthood, suppressed the LPS-induced neurobehavioral changes in the offspring, similar to the symptoms of autism spectrum disorder. The detailed histopathological and immunochemical assays revealed that EMIQ ameliorates the pro-inflammatory and oxidative responses caused by LPS that lead to brain damage.

### 6.5. Musculotropic Effects

Oxidative stress is one of the causes of aging and, among others, may result in muscle atrophy. Thus, the effect of EMIQ supplementation (36 mg/day/kg) on the physiological changes of skeletal muscles related to age was investigated in old mice. Six months of EMIQ consumption were ineffective for age-related muscle loss; however, this significantly improved locomotor activity. As muscle is a critical organ in regulating the metabolism of the whole body, a metabolic profile under sitting and exercise conditions was also determined. Chronic administration of EMIQ prevented the age-related decrease in lipid oxidation and slightly improved the antioxidant capacity and oxidative stress in skeletal muscle, increasing the expression of genes for the antioxidant enzyme glutathione peroxidase and reducing the level of carbonyl proteins [66].

On the other hand, in the study by Kohara et al. [65], EMIQ (4 mg/kg/day) impacted muscle growth in functionally overloaded mice during a 3-week treatment. The overload initiated by the ablation of gastrocnemius and soleus muscles induced hypertrophy of the plantaris muscle. The effect was enhanced by EMIQ treatment alone and in combination with whey proteins. Moreover, muscle hypertrophy was also observable in sham-operated mice.

Evidence for muscle hypertrophy induced by EMIQ was also found in humans. In the study by Omi et al. [50], the effect of EMIQ consumption on the body composition and antioxidant status of men (18.5–30 years old), who regularly undergo intense exercise, was assessed. In the randomized, double-blind, placebo-controlled study, 40 participants took part. One group ate 20 g of supplemental whey protein powder with 42 mg EMIQ, and the other consumed whey protein only (six-times a week, immediately after practice for 4 months). The obtained results suggested that EMIQ supplementation may optimize the effect of exercise and increase muscle hypertrophy. The effects were evidenced by a significant increase in lower limb fat-free mass and muscle mass, exceeding the impact of the training alone. The authors suggested that the results may be related to the antioxidant properties of EMIQ as improvement in antioxidant status was observed. Other mechanism are, however, possible, and require further investigation.

## 7. Methods

The present literature review was conducted by searching the PubMed, Web of Science, Google Scholar, and Science Direct databases using the term “Enzymatically modified isoquercitrin/EMIQ/AGIQ/α-glycosyl isoquercitrin/α-oligoglucosyl quercetin 3-O-glucoside” as descriptors. The inclusion criteria to collect the literature for this review were: (a) articles written in English and published in peer-reviewed journals; (b) articles that reported the manufacturing method, structural aspects, and physicochemical properties of EMIQ; (c) articles that assessed or reported the pharmacological/bioactive effects of EMIQ; (d) articles that reported the safety data of EMIQ; (e) articles that assessed the metabolism and bioavailability of EMIQ. A total of 57 research articles were extracted; the selection was based on their title, abstracts, and keywords. The oldest publications were from 1999, and the most recent one was from August 2022.

## 8. Conclusions

EMIQ is an enzymatically transformed mixture of isoquercitrin α-oligoglucosides with improved bioavailability compared to natural dietary flavonoids such as quercitrin, isoquercitrin, or rutin. It is also characterized by enhanced water solubility, stability, and thermal resistance, which make it particularly suited for food and nutraceutical purposes. EMIQ has already been approved as an antioxidant food additive in the USA and Japan. As the bioactivity data show, it has also considerable potential as a constituent of dietary supplements and nutraceuticals with health-benefiting properties. The conducted animal research demonstrates that EMIQ is a safe product with cardioprotective, anti-allergic, chemopreventive, neuroprotective, and musculotropic properties. In our estimation, EMIQ is an innovative substance with high applicability potential; however, there is still research to be performed before its broader use in nutrition and pharmacy. More clinical trials with human subjects are required to explain in detail the possibilities and limitations of EMIQ’s health effects. It would be undoubtedly interesting to also perform further investigation into the metabolic fate of EMIQ in human intestines, which would broaden the general knowledge on flavonol’s absorption mechanisms.

The case of EMIQ suggests also that α-glucosylation is indeed a very effective method for the bioavailability increase of quercetin. Thus, in our opinion, the development of similar transformation procedures for other valuable phenolics might be worthwhile and constitute an important future direction in plant metabolite supplementation that is beneficial for human health.

## Figures and Tables

**Table 1:** EMIQ metabolites detected in plasma after oral administration and their pharmacokinetic parameters.

Model	Treatment	Detected Metabolites	T_max_	C_max_	AUC	Reference
Male Wistar ST rats	50 µmol/kg p.o.	quercetin conjugates tamarixetin conjugates isorhamnetin conjugates	15 min 15–30 min 6 h	10.7 µM 1 µM 4.8 µM	46.0 µMh 11.2 µMh 55.1 µMh	[10]
ddY mice	4 mmol/kg, p.o.	isoquercitrin quercetin glucuronide quercetin	30 min ^a^	600 µM 95 µM n.d.	- - -	[18]
Harlan Sprague-Dawley rats	1000 mg/kg p.o.	quercetin quercetin glucuronide isoquercitrin	1 h 1 h -	1.0 µg/mL 9.7 µg/mL <LOQ	3.3 h·µg/mL 3.3 h·µg/mL -	[45]
Göttingen minipigs	1000 mg/kg p.o.	quercetin isoquercitrin quercetin glucuronide	30 min–2 h 30 min–2 h 30 min–2 h	0.24 µg/mL 0.13 µg/mL 0.59 µg/mL	0.60 h·µg/mL 0.42 h·µ/mL 2.95 h·µg/mL	[46]
Human volunteers	2 mg aglycone equivalent/kg p.o.	quercetin 3-glucuronide	1.8 h ^b^	1.84 µM ^b^	5.99 µM ^b^	[13]
		quercetin 3′-sulphate				
		isorhamnetin 3-glucuronide	~1.5 h	~0.75 µM	-	

^a^ Time of sample collection (T_max_ not established); ^b^ parameter calculated for the sum of quercetin conjugates; n.d., not detected.

## Data Availability

Not applicable.

## Abbreviations

4-HNE	4-hydroxy-2-nonenal
β3-AR	beta-3 adrenergic receptor
8-OHdG	8-hydroxydeoxyguanosine
ACC	acetyl-CoA carboxylase
*Aldh1a1*	aldehyde dehydrogenase family 1 member A1
AGIQ	alpha-glycosyl isoquercitrin
ALP	alkaline phosphatase
AMP	adenosine monophosphate
AMPK	5′AMP-activated protein kinase
apoE	apolipoprotein E
AUC	area under the plasma concentration–time curve
BHT	butylated hydroxytoluene
BrdU	5-bromo-20-deoxyuridine
BUN	blood urea nitrogen
CAR	constitutive androstane receptor
CD3	cluster of differentiation 3
*Chrm2*	cholinergic receptor, muscarinic 2
*Chrna7*	cholinergic receptor nicotinic alpha 7 subunit
C_max_	maximum plasma concentration
*Cox2*	cyclooxygenase-2; prostaglandin-endoperoxide synthase 2
COXIV	cytochrome c oxidase subunit 4
Cpd	compound
CPT1	carnitine palmitoyltransferase 1
*Cyp1a1*	cytochrome P450, family 1, subfamily a, polypeptide 1
*Cyp2b2*	cytochrome P450, family 2, subfamily b, polypeptide 2
DEN	*N*-diethylnitrosamine
DMF	*N,N*-dimethyl formamide
DR5	death receptor 5
DSS	dextran sulphate sodium
ED2	liver macrophages of the ED2 phenotype
EFSA	European Food Safety Authority
ERK	extracellular signal-regulated protein kinase
EMIQ	enzymatically modified isoquercitrin
FAS	fatty acid synthase
FOS	Fos proto-oncogene
GFAP	glial fibrillary acidic protein
GLP-1	glucagon-like peptide-1
GLUT4	glucose transporter type 4
GMP	good manufacturing practices
*Gpx*	glutathione peroxidase
GRAS	generally recognized as safe
*Grin2d*	glutamate ionotropic receptor NMDA type subunit 2D
*Gstm1*	glutathione S-transferase, mu 1
GST-P	glutathione S-tranferase placental form
γ-GTP	gamma-glutamyl transpeptidase
HO-1	heme oxygenase-1
HOMA-IR	Homeostatic Model Assessment-Insulin Resistance; homeostatic model assessment for insulin resistance
*Iba1*	ionized calcium-binding adapter molecule 1
IFN-γ	type II interferon
IgE	immunoglobulin E
IL	interleukin
IQ	isoquercitrin
IQG	isoquercitrin with α-glucose moiety
IQG*_n_*	isoquercitrin win *n* α-glucose moieties
JAK	Janus kinase
LD_50_	median lethal dose
LPH	lactase-phlorizin hydrolase
LPS	lipopolysaccharide
*Kif21b*	kinesin family member 21B
MAPK	mitogen-activated protein kinase
*Mapk8*	mitogen-activated protein kinase 8
*Mapk14*	mitogen-activated protein kinase 14
*Mapkapk3*	mitogen-activated protein kinase-activated proteinkinase 3
MCAD	medium-chain acyl-CoA dehydrogenase
MGAM	mucosal maltose-glucoamylase
*Me1*	malic enzyme 1
MN-RET	micronucleated reticulocyte(s)
*Mrp2*	multidrug resistance-associated protein 2; ATP-binding cassette, subfamily C, member 2
NADPH	reduced form of nicotinamide adenine dinucleotide phosphate
NEFA	non-esterified fatty acid
*Nfkbia*	nuclear factor of kappa light polypeptide gene enhancer in B-cells inhibitor, alpha
NOAEL	no-observed-adverse-effect level
NOX	NADPH oxidase
*Ntrk2*	neurotrophic receptor tyrosine kinase 2
OECD	Organisation for Economic Cooperation and Development
p.o.	per os; oral administration
PCNA	proliferating cell nuclear antigen
p-ERK1/2	phospho-ERK1/2
PGC-1α	PPAR-γ coactivator 1α
PPARα	peroxisome proliferator-activated receptor α
PRDM 16	PR-domain containing protein 16
QU	quercetin
ROS	reactive oxygen species
RT	rutin
Serpine1	serine (or cysteine) peptidase inhibitor, clade E, member 1
SGLT 1	sodium-dependent glucose transporter 1
SHR	spontaneously hypertensive rat
*Slc17a6*	solute carrier family 17 member 6
SREBP1	sterol regulatory element-binding protein 1
SSZ	sulfasalazine
STAT	signal transducer and activator of transcription proteins
TBARS	thiobarbituric acid reactive substances
TGF-β	transforming growth factor β
T_max_	time to reach C_max_
TNF-α	tumor necrosis factor α
*Tnfrsf10b*	tumor necrosis factor receptor superfamily, member 10b
*Tp53*	tumor protein p53
TUNEL	terminal deoxynucleotidyl transferase-mediated nick end labelining
*Txn1*	thioredoxin 1
UCP 1	uncoupling protein 1
UDP	uridine 5′-diphosphate
U.S. FDA	The United States Food and Drug Administration
UV-C	ultraviolet C light
VCO_2_	carbon dioxide production
VO_2_	oxygen consumption
WRS	water-restraint stress
Yc2	glutathione S-transferase Yc2 subunit
